# Histopathological effects of the fruit extract of *Citrullus colocynthis* on the ovary of the tick *Hyalomma dromedarii*

**DOI:** 10.1007/s10493-023-00895-z

**Published:** 2024-02-12

**Authors:** Asmaa Ali Baioumy Ali, Ashraf Ahmed Montasser, Salma Nabil Ahmed Mohamed

**Affiliations:** Zoology Department, Faculty of Science, Ain Shams University, Abbassia, Cairo, 11566 Egypt

**Keywords:** *Citrullus*, Extract, Histology, *Hyalomma*, Ovary

## Abstract

*Hyalomma dromedarii* is the predominant tick species parasitizing camels in Egypt which leads to mortalities in young animals that result in economic losses. It can transmit a lot of pathogens to animals and humans, such as the Crimean-Congo hemorrhagic fever virus, the Dhori virus, Kadam virus, *Theileria annulata* and spotted fever rickettsia. The continuous use of chemical acaricides has negative impact on the environment and almost led to acaricidal resistance, and hence the plant extracts represent alternative methods for controlling ticks. The present study was carried out to assess the histopathological effects on the ovary of fed female *Hyalomma dromedarii* following immersion in the ethanolic extract of fruits of *Citrullus colocynthis* (100 mg/mL). Light, scanning and transmission electron microscopy observations provided evidence that *Citrullus colocynthis* caused extensive damage to oocytes. Destruction of the internal organelles of oocytes, along with delay and/or inhibition of vitellogenesis were demonstrated. This is the first histological study that points to damage in *H. dromedarii* ovaries following treatment with the ethanolic extract of fruits of *C. colocynthis*. The data presented suggest that the plant extract affects the ovary either directly by entering the oocytes and/or indirectly by damaging the gut cells and digestion of blood that interfere with the development of oocytes, so it can be used as a promising agent for tick control.

## Introduction

Ticks are blood-sucking ectoparasites with worldwide distribution, infesting animals and transmitting severe infectious diseases to animals and humans (Abdel Gawad et al. 2023). Ticks and tick-borne diseases (TBDs) are widespread in Egyptian localities (Abdelbaset et al. 2022). *Hyalomma dromedarii* (Ixodoidea: Ixodidae) is the predominant tick species parasitizing camels in Egypt (Abdel-Ghany et al. 2023). It causes mild to severe anemia; annoying bites, anorexia, skin spoilage, intense pruritus, loss of appetite, reduction in growth rate, decreased productivity, and mortalities in young animals that result in economic losses (Guglielmone et al. 2014).

*Hyalomma dromedarii* can transmit many diseases such as Q fever (*Coxiella burnetii*) (Bazlikova et al. 1984), theileriosis (*Theileria camelensis* and *T. annulata*) (Hoogstraal et al. 1981; Mohamed et al. 2021), Crimean-Congo hemorrhagic fever (Bendary et al. 2022), spotted fever (*Rickettsia rickettsii*) (Al-Deeb et al. 2015), and African tick-borne fever (*R. africae*) (Abdel-Shafy et al. 2012; Abdullah et al. 2019). So, its control is very important for the prevention of tick-borne diseases.

The usage of chemical acaricides resulted in contamination of the environment, residues in milk and meat products and the development of acaricide-resistant ticks (Iqbal et al. 2021; Mohammed et al. 2023). Alternative control strategies to overcome the drawbacks associated with acaricides are an urgent need (Mohamed et al. 2022; Eltaly et al. 2023). The use of herbal medications is one of the most promising alternative approaches for the treatment of various infectious agents because of their biodegradability, target efficiency and cost effectiveness (Nugraha et al. 2020; Zheoat et al. 2021; Abdel-Meguid et al. 2022).

The fruits of *C. colocynthis* contain a number of bioactive constituents, including glycosides, flavonoids and alkaloids (Hussain et al. 2022) with anti-inflammatory, anti-diabetic, anti-microbial, anti-helminthic, antibacterial, anti-carcinogenic, anti-ulcerogenic, hypolipidemic, hypoglycemic, and anti-oxidant properties (Ullah et al. 2013; Bhasin et al. 2020). The different extracts of it showed insecticidal activity against different insect species such as *Culex quinquefasciatus* and *Aedes aegypti* (Rahuman et al. 2008; Sakthivadivel and Daniel 2008), *Aphis craccivora* (Dimetry et al. 2015), *Anopheles arabiensis* (Hamid et al. 2016) and *Brevicoryne brassicae* (Ahmed et al. 2020). Its acaricidal efficacy was studied at biological levels only against mites such as *Tetranychus urticae* (Dahroug et al. 2000) and *Rhizoglyph ustritici* (Bashir et al. 2013), and against different stages of ticks; *H. analoticum* (Godara et al. 2015), *Rhipicephalus microplus* (Ullah et al. 2015), *Rhipicephalus* sp. (Hussian and Jasim 2018) and *H. dromedarii* (Mahran et al. 2020; Mohamed et al. 2022).

Many studies were carried out to detect the histopathological effects of plant extracts on the female reproductive system of hard ticks such as *Amblyomma cajennense* (Anholeto et al. 2018), R. *sanguieus* (Souza et al. 2019)d *microplus* (Reis et al. 2021). Although a few authors tested plant extracts against different developmental stages of *H. dromedarii* (Abdel-Shafy et al. 2007; Habeeb et al. 2007; Mahran et al. 2020; Mohamed et al. 2022; Abdel-Ghany et al. 2021a, b, 2023; Eltaly et al. 2023), none of them studied the histology of internal organs. Therefore, this work is aimed at studying the histopathological effect of an ethanolic ectract of the fruits of *C. colothynthis* on the ovary of the tick, *H. dromedarii*.

## Materials and methods

### Tick collection

*Hyalomma dromedarii* ticks were collected from the naturally infested camels at Birqash camel market (30°9′58.4″N, 31°2′13.2″E), Giza Governorate, Egypt. They were identified according to Hoogstraal and Kaiser (1958), and grouped into non-engorged, semi-engorged and engorged males and females according to Stark et al. (2018). The engorged females were kept in glass vials, covered by pieces of gauze securely held by rubber bands, in the incubator at 28 ± 2 °C and 75–80% relative humidity until the experiments were conducted (within 24 h of collection).

### Preparation of the extract

Ripen dried fruits of *Citrullus colocynthis* were bought from the market, cleaned to remove dust, and ground using a stainless steel knife mill. Ethanolic extract was prepared according to the method of Twaij et al. (1988). Plant powder (50 g) was added to 80% ethyl alcohol (250 mL), covered with aluminum foil and kept in dark condition for 72 h, then it was filtrated using Whatman filter paper (110 mm diameter opening). The filtrate was poured in glass petri dishes and put in the incubator at 50 °C for alcohol evaporation. Finally, the dried extract was collected, weighed, transferred to glass vials, and kept at 4 °C until use. A concentration of 100 mg/mL was prepared by dissolving 1 g of the extract in 10 mL of distilled water.

### Treatment

Adult immersion test (AIT) was performed as described by Drummond et al. (1973). Each engorged female tick was immersed in 10 mL of 100 mg/mL concentration for 5 min and transferred to sterile glass vials securely covered by gauze. Treated and untreated ticks were kept in the incubator at 28 ± 2 °C and 75–80% relative humidity.

### Morphological and histological studies

Both untreated and treated ticks were dissected 4 and 7 days after engorgement and treatment, respectively. Thirty-six tick females were dissected throughout the experiments. Three replicates each with three females were kept for the untreated and treated specimens at each period.

### Light microscopy

Female ticks were dissected in a Petri dish containing a mixture of paraffin wax and charcoal and covered by 0.85% NaCl solution under a dissecting binocular microscope. The dorsal integument was removed, and the tick was washed several times with saline solution for blood removal then fixed in Bouin̕̕s fixative for 24 h (Bancroft et al. 2008). Specimens were dehydrated in an ascending series of ethyl alcohol, then transferred to methyl benzoate for 24 h to soften the integument. Samples were then transferred to a solution of 2% celloidin in methyl benzoate for 24 h (Gatenby and Beams 1950), cleared in benzol, infiltrated in three changes of paraplast at 56 °C and then embedded in paraplast (Fisher Scientific Inc. USA).

Serial transverse sections were cut at 3 μm thick using a YD-335 computer microtome (Huran Kaida Scientific Instrument Comp., China), and stained with Mallory triple stain (MT) (Pantin 1946) or hematoxylin-eosin stain (HE) (Pears 1960), then photographed using a Samsung ES95 HD digital camera fixed on an Olympus microscope (Japan made).

### Electron microscopy

Female ticks were dissected in cold phosphate buffer adjusted to pH 7.2, and the ovaries were removed and fixed for 2 h in 3% cold fresh glutaraldehyde. They were washed for 30 min in phosphate buffer.

### Scanning electron microscopy (SEM)

After washing in phosphate buffer, ovaries were dehydrated in ascending series of ethanol. Samples were subjected to critical point drying, attached to aluminum stubs, coated with gold in a sputter-coating apparatus, and then examined and photographed under a Quanta FEG 250 scanning electron microscope (FEI Company, Hillsboro, Oregon, USA) at the Electron Microscope Unit, Desert Research Center, Cairo, Egypt.

### Transmission electron microscopy (TEM)

After washing in phosphate buffer, ovaries were postfixed in cold 1% osmic acid for 2 h and washed again in fresh buffer. They were then dehydrated in ascending series of ethanol and embedded in an epoxy resin (Gatenby and Beams 1950).

Semithin Sections. (500 to 1000 nm) from blocks were prepared using Leica Ultra-cut (UCT ultra-microtome) and stained with toluidine blue stain (TB) (Dawes 1971) for light microscope examination before ultrasectioning. With a sharp diamond knife and the same ultratome, ultrathin Sections. (75–90 nm) were cut, mounted on copper grids (grid size 300 mesh × 83 μm pitch) and stained with uranyl acetate and lead citrate (Reynold, 1963). Oocytes were examined by transmission electron microscope JEOL (JEM-1400 TEM) at the Electron Microscope Unit, Faculty of Agriculture, Cairo University, Egypt.

## Results

**Fig. 1:**
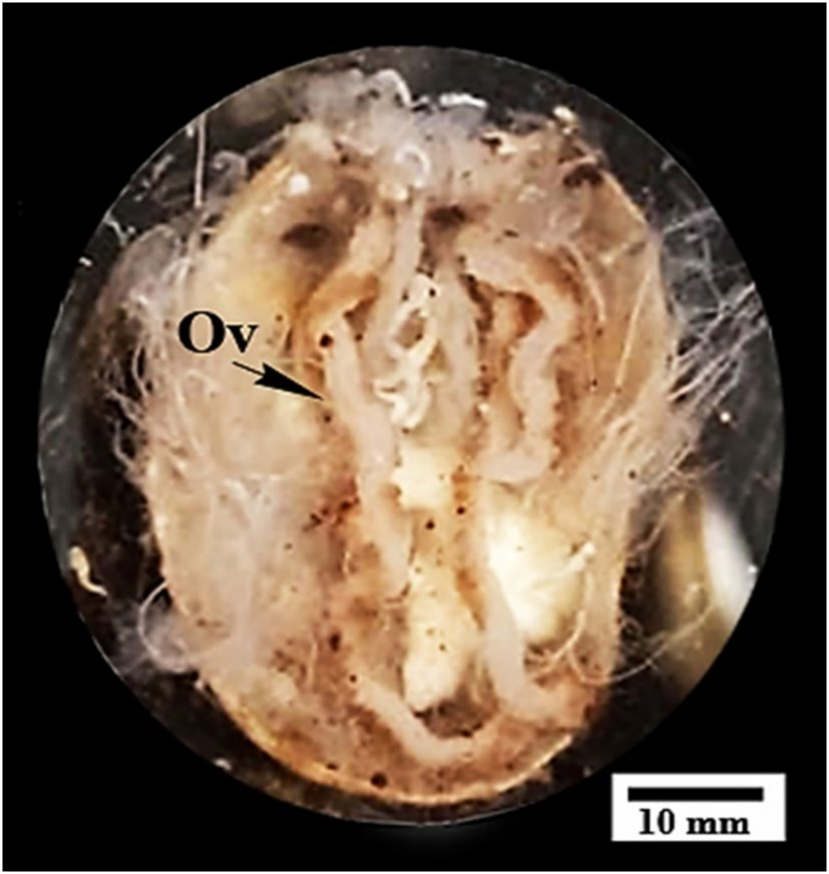
Dissected untreated female *Hyalomma dromedarii* showing the horseshoe shape of the ovary (Ov) and its position in the postero-lateral region of body cavity

The comparison between untreated and treated ovaries changes was summarized in Table 1. The ovary of untreated *H*. *dromedarii* is a tubular structure in the form of a horseshoe located in the postero-lateral region of the body cavity extending approximately in whole body length (Fig. 1).

**Table 1:** The summarize of the comparative histological changes between untreated *Hyalomma dromedarii* ovary and different oocyte stages, and those treated with 100 mg/mL of fruit alcoholic extract of *Citrullus colocynthis*

Item		Control 4th day		Control 7th day	Treated 4th day	Treated 7th day
Gross features of ovary		• Tubular structure, horseshoe in shape (Fig. 1) • Located in the postero-lateral region of body (Fig. 1). • Extending in whole body length (Fig. 1) • A grape-like hollow organ surrounded by a wall (Fig. 3a). • The ovarian wall contains epithelium and oocytes (Fig. 3a)			• Elongated ovary (Fig. 2d) • Presence of fluid-filled spaces at the attachment site with pedicle cells (Fig. 3f)	• Damaged epithelial cells (Fig. 3d) • Deformation of the pedicle cells (Fig. 3d)
Gross features of oocytes		• Protruding into hemolymph (Fig. 3a) • Connected to the ovarian wall with pedicle (Fig. 3a)	• More or less oval in shape (Fig. 2a) • The differentiation of late stages was clearly demonstrated (Fig. 2c) • Protruding from the ovarian surface, with a few number of early ones (Fig. 2c)		• Damaged oocytes (Fig. 2d) • Suppression of oocytes development (Fig. 2e)	• Highly deformed oocytes (Fig. 2f) • Oocytes decreased in number (Fig. 3d)
SEM of ovary		• A grape-like structure with different oocyte stages (I to V) (Fig. 2a and c) • Oocytes more or less oval in shape (Fig. 2a and c). • Oocytes develop asynchronously (Fig. 2a and c)			• Elongation (Fig. 2d) • Damaged oocytes (Fig. 2d)	
		• Presence of a large number of early developing oocytes (I and II) (Fig. 2b) • Stage III of oocytes begin to form (Fig. 2b)	• Late oocyte stages (III to V) protruded from ovarian surface. (Fig. 2c) • Stage V is covered by the eggshell (chorion) (Fig. 2c)		• Suppression of oocytes development (Fig. 2e) • No differentiation between early stages and stage III (Fig. 2e) • Oocytes appeared like fused molten globules (Fig. 2e) • Oocytes had indistinct boundaries (Fig. 2e)	• Highly deformed oocytes (Fig. 2f) • Absence of late stages (Fig. 2f) • Fused early stages in large number (Fig. 2f) • Late stages lost their ovoid shape (Fig. 2f) • Wrinkling or eroding surfaces of late stages (Fig. 2f)
Oocyte I	LM	• Oval to polygonal in shape (Fig. 3a) • Facing the ovarian lumen (Fig. 3a) • Large oval to round nuclei (Fig. 3a)	• Very few in number if found, with the same characters		• Appeared as misshapen and heterogeneous cell masses (Fig. 3d)	• Couldn’t be distinguish as it was completely damaged
	TEM	• Cytoplasm contained mitochondria with free ribosomes (Fig. 4a) • Large oval to round nuclei occupying a wide area of cytoplasm (Fig. 4a) • Low nucleoplasm density is low with few dense heterochromatin (Fig. 4a)	• Very few in number if found, with the same characters		• Couldn’t be distinguish as it was completely damaged	• Couldn’t be distinguish as it was completely damaged
Oocyte II	LM	• Great cytoplasmic growth (Fig. 3a) • Oval in shape (Fig. 3a). • Had dense and granulated cytoplasm (Fig. 3a) • Connect to the ovarian wall by pedicle cells (Fig. 3a)	• Very few in number if found, with the same characters		• Ruptured membranes (Fig. 3e) • Vacuolated karyolysed nuclei (Fig. 3e)	• Great deformities in their shapes (Fig. 3d)
	TEM	• Nucleoplasm is occupied with euchromatin and a large nucleolus (Fig. 4b) • The cytoplasm contains rough endoplasmic reticulum, ribosomes, lipid droplets and small mitochondria (Fig. 4b) • Short microvilli with pedicle cells (Fig. 4b)	• Very few in number if found, with the same characters		• Numerous vacuoles in the cytoplasm (Fig. 4c) • Nuclei without heterochromatin (Fig. 4c) • The nucleolus was relatively large (Fig. 4c)	• Vacuolation increased (Fig. 4d). • Vacant space inside nucleolus (Fig. 4d).
Oocyte III	LM	• Become virtually visible (Fig. 3a) • Early appearance of yolk granules (Fig. 3a)	• Early formation of the eggshell (chorion) (Fig. 3b) • The nucleus is not easily detected (Fig. 3b)		• Pycnotic nuclei with ruptured membrane (Fig. 3e) • Wrinkled boundaries (Fig. 3f). • Great deformities in shape (Fig. 3f) • The cytoplasm condensed with vacuoles (Fig. 3f)	• Appeared with marked alterations and highly damaged (Fig. 3d)
	TEM	• Well-developed cytoplasmic organelles (Fig. 4e) • Yolk originated from small vesicles derived from Golgi bodies and rough endoplasmic reticulum, shared together to form larger multi-vesicular bodies (Fig. 4e) • Few lipid droplets (Fig. 4e)	• A layer of tunica propria was clearly observed (Fig. 4f) • The eggshell was very thin shows few microvilli (Fig. 4f)		• Folded nuclear membrane (Fig. 4g) • Squashing of nucleoplasm and chromatin granules (Fig. 4g) • Vacuolated nucleolus (Fig. 4g) • Lysis of cell organelles (Fig. 4h) • Presence of cytoplasmic vacuoles (Fig. 4h) • Yolk granules were rarely appeared (Fig. 4h). • Damaged microvilli (Fig. 4h)	• Vacuolation increased in the cytoplasm (Fig. 4i) • Irregular or crescent-like shape yolk granules appeared irregular (Fig. 4i) • Yolk granules decreased in number (Fig. 4i) • Abnormal formation of eggshell (Fig. 4i)
Oocyte IV	LM	• Still not formed	• Oval in shape (Fig. 3a) • Large yolk granules (Fig. 3a) • Increasing eggshell deposition (Fig. 3a)		• Still not formed	• Irregular in shape (Fig. 3g) • Condensed or squashed cytoplasmic area (Fig. 3g) • Presence of cytoplasmic vacuolation (Fig. 3 g and 3 h) • Deformed yolk granules (Fig. 3h). • Chorion disruption (Fig. 3h)
	TEM	• Still not formed	• Contain large electron dense homogeneous yolk granules fill most of cytoplasm (Fig. 5a and b) • Presence of Golgi bodies and mitochondria (Fig. 5a and b) • Lipid droplets increased in size and quantity (Fig. 5a and b) • Increase in eggshell deposition resulted in stretching of the tunica propria (Fig. 5b)		• Still not formed	• Damage or disappearance of cytoplasmic organelles (Fig. 5c) • Fragmented yolk granules (Fig. 5c) • abnormal or defect in eggshell formation (Fig. 5d) • Separation between cell membrane and tunica propria (Fig. 5d) • Damaged microvilli (Fig. 5d)
Oocyte V	LM	• Still not formed	• Became clearly visible (Fig. 3c) • Contain very large yolk granules (Fig. 3c) • Well-developed and very thick eggshell (Fig. 3c)		• Still not formed	• Couldn’t be observed
	TEM	• Still not formed	• Containing fully formed large yolk granules completely filled the cytoplasm (Fig. 5e) • The microvilli reached their maximum length (Fig. 5e) • Stretched tunica propria (Fig. 5e) • Very thick eggshell (Fig. 5e)		• Still not formed	• Couldn’t be observed

**Fig. 2:**
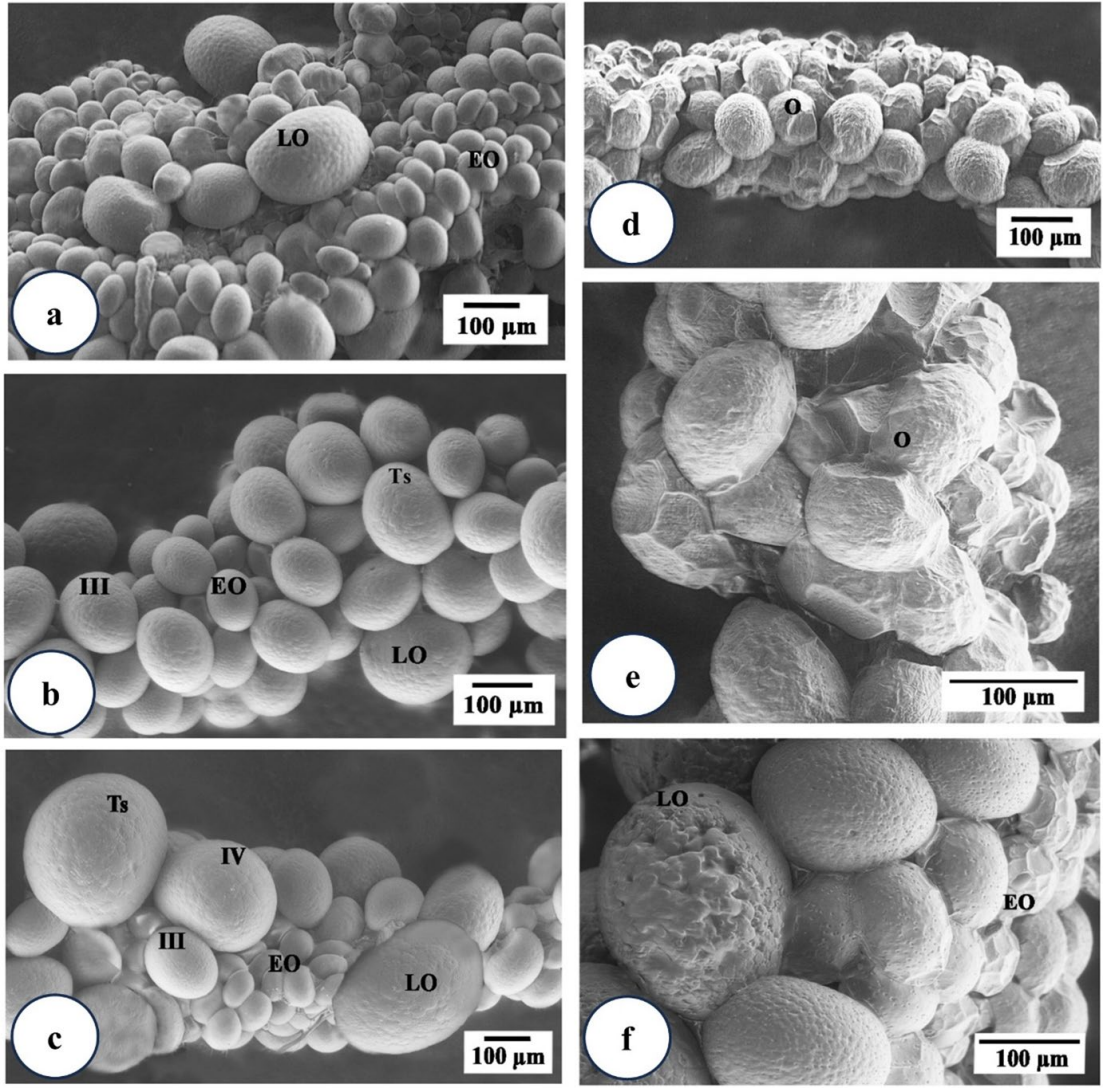
Scanning electron micrographs of *Hyalomma dromedarii* ovary. **a** 7 days after engorgement showing its grape-like appearance containing early (EO) and late (LO) stages of oocytes. **b** 4 days after engorgement showing early (EO) and first stage (III) of late oocytes (LO) with tuberculated surfaces (Ts). **c** 7 days after engorgement showing early (EO) and late stages (III and IV) of developing oocytes with tuberculated surfaces (Ts). **d** 4 days after treatment showing deformities in the shape of oocytes (O). **e** 4 days after treatment showing oocytes (O) appeared as fused molten globules with indistinct boundaries. **f** 7 days after treatment showing higher magnification of damaged early (EO) and late oocytes (LO) with fused boundaries and wrinkled surfaces

Scanning electron microscopical examination (SEM) revealed that the ovary appears as a grape-like structure (Fig. 2a and c). It contains numerous oocytes at different developmental stages, from early (I and II) to late stages (III, IV, and V), (Fig. 2b and c). Oocytes are more or less oval in shape and develop asynchronously (Fig. 2a and c).

Four days after engorgement, the late stages of oocytes begin to form, especially stage III, in the presence of a large number of early developing ones (Fig. 2b). The differentiation of late stages was clearly demonstrated as protruding from the ovarian surface, with a few early ones after seven days of engorgement (Fig. 2c). The outer surface of more developed oocytes (Stage V) is completely covered by a rough tuberculated surface called the eggshell (chorion) (Fig. 2c).

The ovary of the treated female *H*. *dromedarii* showed elongation with damaged and deformed oocytes (Fig. 2d). It was observed that after 4 days of treatment, the development of oocytes was markedly suppressed, and it was difficult to differentiate between early stages and stage III (Fig. 2e) when compared with the untreated ones. Oocytes appeared like fused molten globules with indistinct boundaries (Fig. 2e).

Seven days after the treatment, the ovary showed highly deformed oocytes with no late stages of oocytes (Fig. 2f). Some oocytes were fused together with no distinct boundaries between them, especially the early stages that found in large number (Fig. 2f). Scanning electron microscopical examination of late stages of oocytes revealed signs of damage represented by loss of their ovoid shape and wrinkling or eroding of their surfaces (Fig. 2f).

**Fig. 3:**
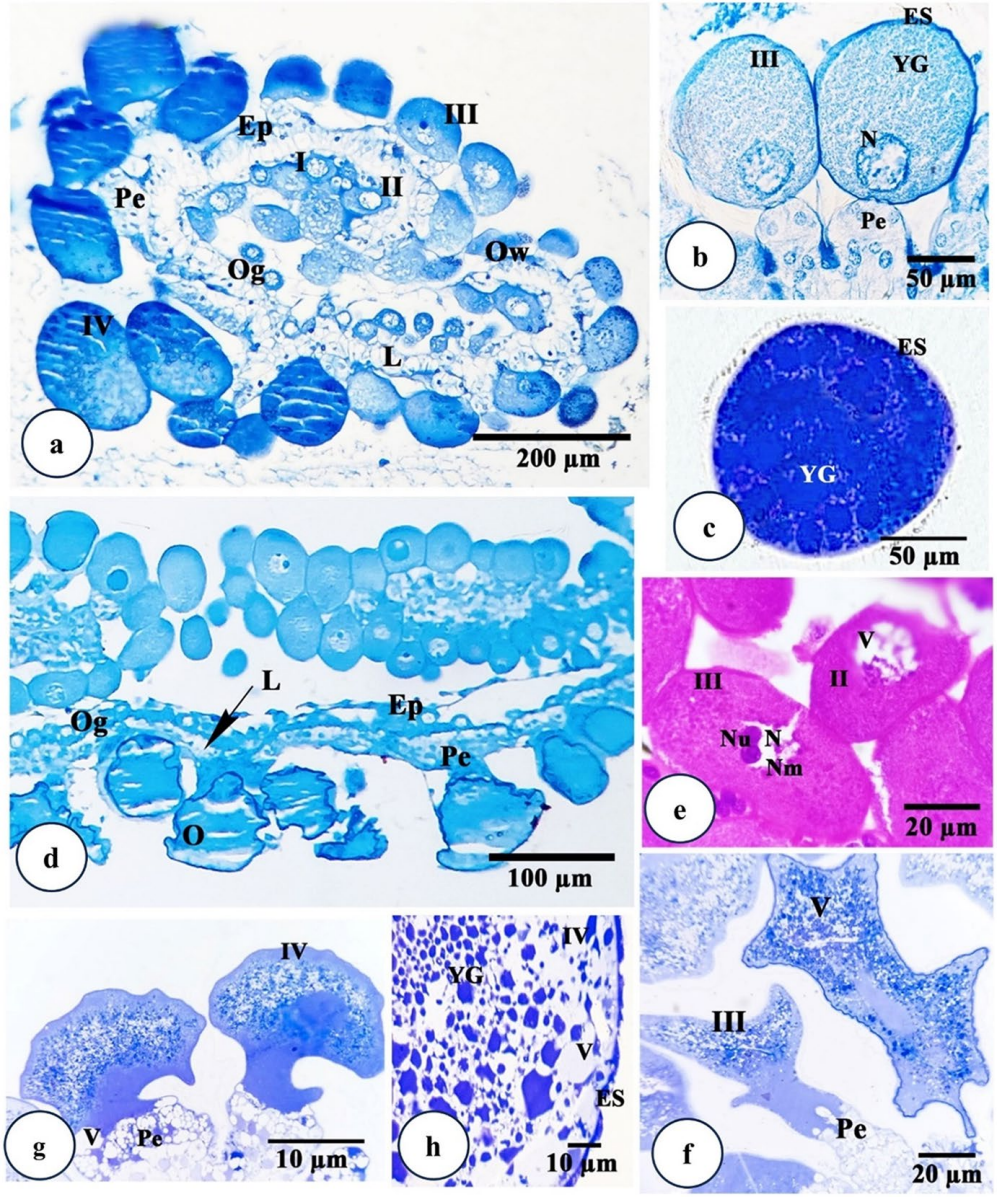
Light micrographs of transverse sections of *Hyalomma dromedarii* ovary. **a** Paraffin Section 4 days after engorgement showing protruding oocytes giving it the grape-like appearance, ovarian epithelium (Ep), pedicle cells (Pe) and ovarian lumen (L). I: Oocyte stage I. II: Oocyte stage II, III: Oocyte stage III, IV: Oocyte stage IV, Og: Oogonia, Ow: Ovarian wall. MT stain. **b** Paraffin Section 7 days after engorgement showing oocyte stage III (III) with almost rounded nucleus (N), beginning of yolk granules (YG) formation, eggshell (ES) in its early stage and the connection to pedicle cells (Pe). MT stain. **c** Paraffin Section 7 days after engorgement showing oocyte stage V with fully formed yolk granules (YG) and covered with eggshell (ES) (chorion). MT stain. **d** Paraffin Section 7 days after treatment showing marked alterations in the ovarian epithelium (Ep), oocytes (O) of various developmental stages and pedicle cells (Pe). L: Lumen, Og: Oogonia. MT stain. **e** Paraffin Section 4 days after treatment showing higher magnification of oocyte stages II (II) and III (III) with vacuolation of nucleus (N) and abnormal shape of nucleoulus (Nu) in addition to damage of nuclear membrane (Nm). V: Vacuole. HE stain. **f** Semithin Section 4 days after treatment showing deformed oocyte stage III (III) with wrinkled boundaries, coalesced cytoplasm and deformed pedicle cell (Pe) with vacuoles (V). TB stain. **g** Semithin Section 7 days after treatment showing oocyte stage IV (IV) with large squashed cytoplasmic areas at its periphery and extensively damaged pedicle cells (Pe) containing numerous vacuoles (V). TB stain. **h** Semithin Section 7 days after treatment showing oocyte stage IV (IV) with damaged yolk granules (YG), cytoplasmic vacuoles (V) and disrupted eggshell (ES). TB stain

Examination of the *H. dromedarii* ovary under a light microscope revealed a grape-like hollow organ enclosing a lumen surrounded by a thin ovarian wall (Fig. 3a). This wall consisted of epithelial cells, oogonia and different oocyte stages protruding into hemolymph and connected to the ovarian wall by a thin stalk, the pedicle (Fig. 3a).

Oogonia were very small in size, oval in shape, appeared in densely packed clusters (Fig. 3a). They were characterized by their high nucleocytoplasmic ratio (Fig. 3a). Stage I oocytes resulted from the oogonial division. They were oval to polygonal in shape and faced the ovarian lumen (Fig. 3a). Their cytoplasm showed large oval to round nuclei that occupied a wide area. Stage II oocytes were characterized by great cytoplasmic growth. It’s formation extends until the beginning of yolk granules formation then changes into the next stage. They appeared oval in shape with dense and granulated cytoplasm that was connected to the ovarian wall by pedicle cells (Fig. 3a).

Stage III oocytes were virtually visible four days after engorgement and characterized by the appearance of yolk granules in addition to the early formation of the egg shell (chorion) (Fig. 3a and b). The nucleus was not easily detected and if so, it appeared almost rounded with dispersed euchromatin and a relatively large, rounded nucleolus (Fig. 3a and b). Stage IV oocytes were oval in shape (Fig. 3a). It contained large yolk granules that fill most of the cytoplasm with increasing eggshell deposition indicated by high intensity of staining (Fig. 3a). Stage V oocytes became clearly visible seven days after engorgement, containing fully formed yolk granules that became very large and completely filled the oocyte cytoplasm (Fig. 3c). The eggshell is well developed and very thick.

Generally, marked alterations in epithelial cells, oocytes and pedicle cells were noticed after treatment. The number of oocytes decreased, and the remaining ones were damaged (Fig. 3d). Oogonia and stage I oocytes were completely damaged and appeared as misshapen and heterogeneous cell masses (Fig. 3d). Four days after treatment, stage II oocytes showed ruptured membranes with vacuolated karyolysed nuclei (Fig. 3e). On the other hand, stage III oocytes showed pycnotic nuclei with ruptured membrane (Fig. 3e). Some of these oocytes exhibited wrinkled boundaries with great deformities in their shapes (Fig. 3f). The cytoplasm coalesced, shrunk, and condensed in some areas, with vacuoles (Fig. 3f).

Seven days after treatment, oocyte IV appeared irregular in shape and highly damaged with coalesced cytoplasm (Fig. 3g). A large squashed cytoplasmic area at the periphery of the oocytes was observed (Fig. 3g). Fluid-filled spaces were detected at the attachment site of pedicle cells. Cytoplasmic vacuolation was observed (Fig. 3g and h). Yolk granules were highly deformed with chorion disruption in some areas (Fig. 3h).

**Fig. 4:**
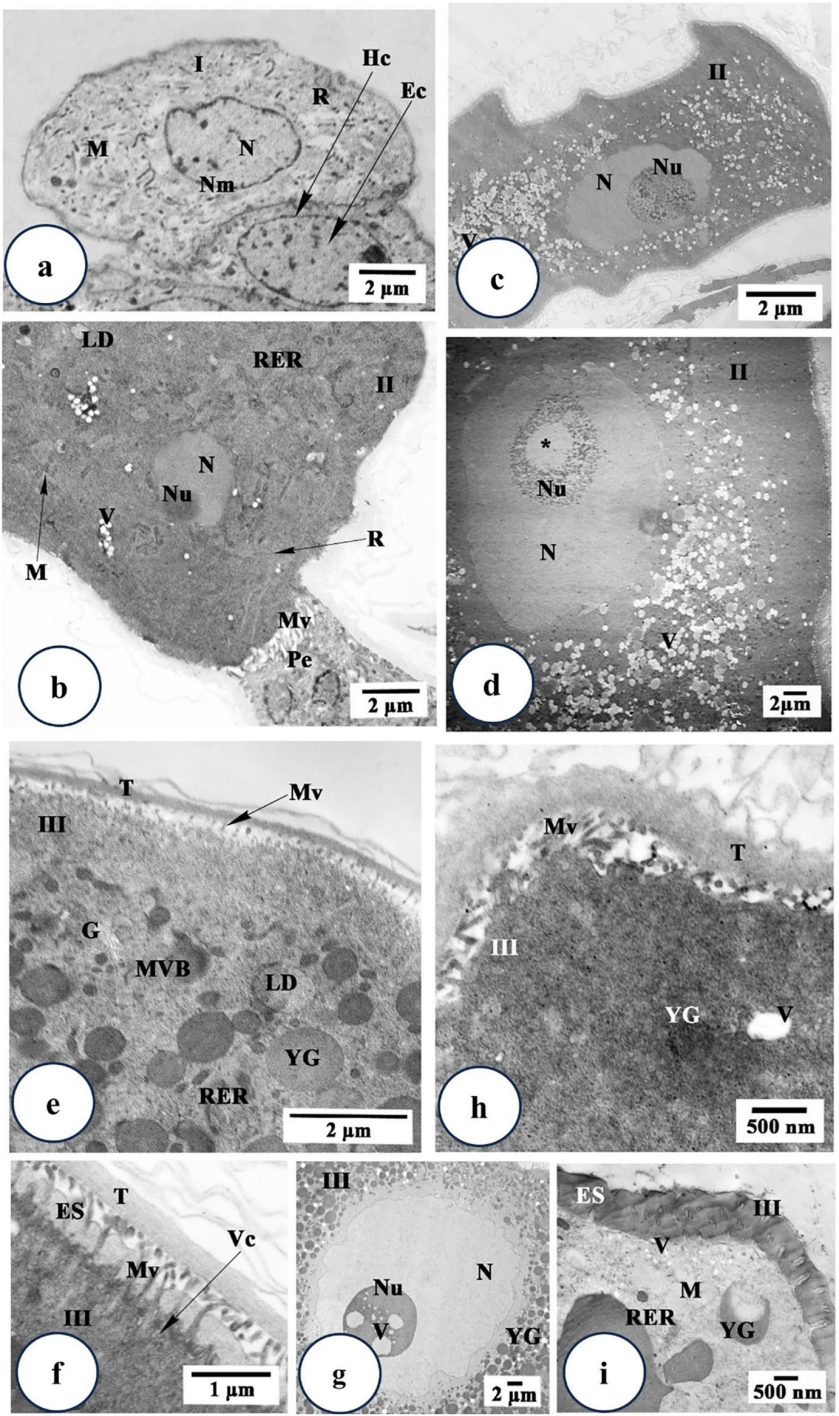
Transmission electron micrographs of stages I, II and III oocytes from *Hyalomma dromedarii* ovary. **a.** stage I (I) oocyte 4 days after engorgement showing nucleus (N), mitochondria (M), ribosomes (R) and low density nucleoplasm with euchromatin (Ec) and few dense masses of heterochromatin (Hc) beneath the nuclear membrane (Nm). **b** Stage II (II) oocyte 4 days after engorgement showing nucleus (N), nucleolus (Nu), rough endoplasmic reticulum (RER), ribosomes (R), mitochondria (M), lipid droplets (LD) and the connection to pedicle cells (Pe) with microvilli (Mv). **c** Stage II (II) oocyte 4 days after treatment showing great deformation of its shape and presence of numerous vacuoles (V) in the cytoplasm. Nu: Nucleolus, N: Nucleus. **d.** Stage II (II) oocyte 7 days after treatment showing cytoplasm with numerous vacuoles (V), abnormal nucleus (N) and damaged nucleolus (Nu) with vacant space (astrisk). **e** Stage III (III) oocyte 4 days after engorgement showing the presence of tunica propria (T), microvilli (Mv) and cytoplasm with Golgi complex (G), rough endoplasmic reticulum (RER), lipid droplets (LD), multi-vesicular bodies (MVB) and small electron dense homogeneous yolk granules (YG). **f** Stage III (III) oocyte 7 days after engorgement showing the presence of thin eggshell (ES) below the tunica propria (T). Mv: Microvilli. Vc: Vesicle. **g** Stage III (III) oocyte 4 days after treatment showing irregular nucleus (N), nucleolus (Nu) with vacuoles (V) and many yolk granules (YG). **h** Stage III (III) oocyte 4 days after treatment showing damaged microvilli (Mv), aggregated areas of cytoplasm at the periphery of oocyte and vacuoles (V) in the cytoplasm. T: Tunica propria, YG: Yolk granules. **i** Stage III (III) oocyte 7 days after treatment showing lysed mitochondria (M), irregular and crescent shape yolk granules (YG) and vacuoles (V) at the periphery of the oocyte. ES: Eggshell, RER: Rough endoplasmic reticulum

Using TEM, cytoplasm of stage I oocytes of untreated ticks appeared contained mitochondria with free ribosomes and large oval to round nuclei that occupy a wide area of the cytoplasm (Fig. 4a). The nucleoplasm density is low exhibiting mainly euchromatin with few dense masses of heterochromatin beneath the nuclear membrane (Fig. 4a). After treatment with the extract, it was difficult to distinguish this stage as it was completely damaged.

Transmission electron microscopical examination of stage II oocyte reveals that the nucleoplasm is occupied with euchromatin and a prominently large nucleolus (Fig. 4b). Their cytoplasm contained numerous rough endoplasmic reticulum, ribosomes, lipid droplets and small mitochondria (Fig. 4b). Short microvilli appeared between this oocyte stage and pedicle cells (Fig. 4b).

Four days after treatment, numerous vacuoles in the cytoplasm of stage II oocytes were observed (Fig. 4c). Their nuclei were without heterochromatin and the nucleolus was relatively large (Fig. 4c). Vacuolation increased seven days after treatment with the presence of vacant space inside nucleolus (Fig. 4d).

In the stage III oocytes, the cytoplasmic organelles were well developed (Fig. 4e). A layer of tunica propria, that separates the ovarian tissue from the haemolymph, was clearly observed (Fig. 4e and f). Yolk originated from small vesicles derived from Golgi bodies and rough endoplasmic reticulum, joined together to form larger multi-vesicular bodies (Fig. 4e). Through repeated fusions between these bodies, electron dense homogeneous yolk spheres are formed. Lipid droplets were observed in few numbers (Fig. 4e). Deposition of eggshell began slightly later to the beginning of yolk formation. Eggshell (chorion) developed from vesicles, derived from Golgi bodies, which reached cell membrane and fuse with it and/or microvilli discharging their content (exocytosis) into the extracellular space between the plasma membrane and the tunica propria (Fig. 4f). The eggshell at this stage was very thin below the tunica propria and the surface of the oocyte membrane showed few microvilli (Fig. 4e and f).

Transmission electron microscopical examination of treated ticks after 4 days displayed folded nuclear membrane and squashing of nucleoplasm and chromatin granules of stage III oocyte (Fig. 4g). Nucleolus contained numerous vacuoles (Fig. 4g). A lysis of cell organelles such as mitochondria and rough endoplasmic reticulum was noticed (Fig. 4h). The presence of cytoplasmic vacuoles and yolk granules were rarely appeared (Fig. 4h). Seven days after treatment, vacuolation increased in the cytoplasm (Fig. 4i). Yolk granules appeared irregular or crescent-like shape and decreased in number (Fig. 4i). There was marked damage in the oocytes’ microvilli (Fig. 4h) that led to abnormal formation of eggshell (Fig. 4i) after seven days of treatment.

**Fig. 5:**
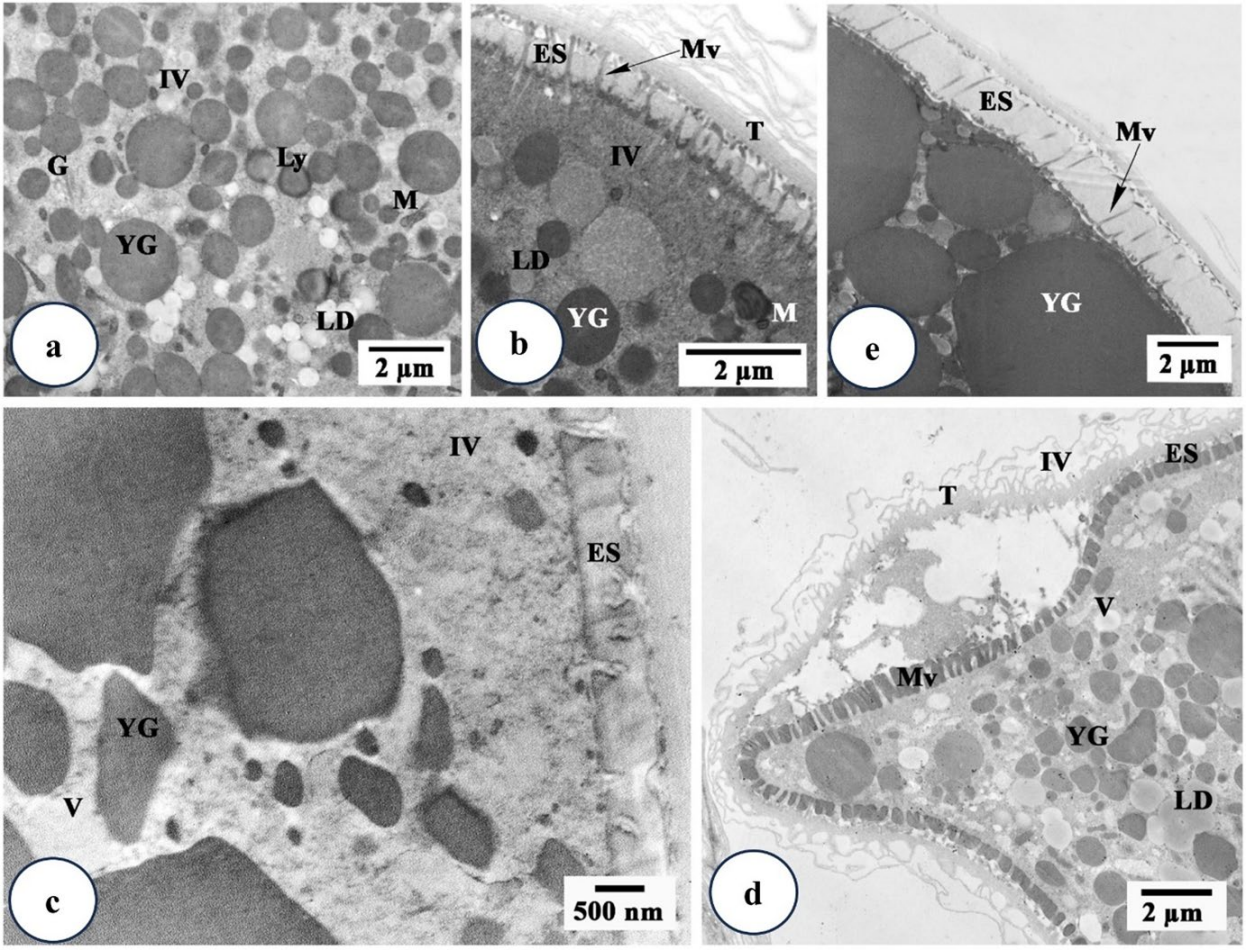
Transmission electron micrographs of stages IV and V oocytes from *Hyalomma dromedarii* ovary. **a** stage IV (IV) oocyte 7 days after engorgement showing its cytoplasm with large electron dense yolk granules (YG), Golgi complex (G), mitochondria (M) and lipid droplets (LD). Ly: Lysosomes. **b** Stage IV (IV) oocyte 7 days after engorgement showing the increase in eggshell (ES) deposition that resulted in stretching of tunica propria (T). LD: Lipid droplets, M: Mitochondria, Mv: Microvilli, YG: Yolk granules. **c** Stage IV (IV) oocyte 7 days after treatment showing deformed and lysed yolk granules (YG) and abnormal eggshell (ES). V: Vacuoles. **d** Stage IV (IV) oocyte 7 days after treatment showing the separation between the eggshell (ES) and tunica propria (T) and damaged microvilli (Mv). LD: Lipid droplets, V: Vacuoles, YG: Yolk granules. **e** Stage V oocyte 7 days after engorgement showing the fully formed yolk granules (YG) in the cytoplasm and the thick eggshell (ES). Mv: Microvilli

Stage IV oocytes were markedly observed seven days after engorgement in untreated tick. Each contained large electron dense homogeneous yolk granules which fill most of the oocyte cytoplasm (Fig. 5a and b). Some Golgi bodies and mitochondria were observed between yolk granules. Lipid droplets markedly increased in size and quantity (Fig. 5a and b). Obvious increase in eggshell deposition resulted in stretching of the tunica propria that appeared with fewer fibers (Fig. 5b).

Transmission electron microscopical examination of these oocytes showed great damage or disappearance of cytoplasmic organelles (Fig. 5c). There was deformation of yolk granules, and some were fragmented (Fig. 5c). The extract caused distinct separation between cell membrane and tunica propria in addition to damaged microvilli (Fig. 5d) leading to abnormal or defect in eggshell formation.

Stage V oocytes became clearly visible seven days after engorgement. It is characterized by containing fully formed large yolk granules completely filled the cytoplasm (Fig. 5e). The microvilli reached their maximum length and appeared as straight tubes with stretched tunica propria and the eggshell became very thick (Fig. 5e).

Stage V oocytes could not be observed after treatment in the ovaries of females during the examined period.

## Discussion

The female genital system is the most complicated system in the ixodid tick body (Balashov 1972). In the present study based on microscopical examination of untreated female *Hyalomma dromedarii*, there was a single, tubular, U-shaped ovary lying in the posterior half of the body. Similar to that described in other ticks (Balashov 1972, 1983), ovary appeared as a hollow organ enclosing a lumen surrounded by a thin ovarian wall that was composed of epithelial cells, oogonia, and oocytes in various developmental stages. An outer tunica propria that separates the ovarian tissue from the hemolymph was also observed. After feeding and mating, oocytes protrude into the haemocoel, giving the ovary a grape-like appearance, with oocytes attached to the ovarian wall through pedicles. The ovary then increases in size considerably exhibiting eggs (Rey 1973) until they are ready for oviposition (Oliveira et al. 2006).

Oocytes in the present study might be classified into oogonia and stages I, II, III, IV and V based on Roshdy (1969) and Balashov (1983) classifications. Similarly, oocytes were classified into five stages in *Haemaphysalis longicornis* (Yano et al. 1989; Mihara et al. 2018), *Amblyomma cajennense* (Denardi et al. 2004), *Rhipicephalus microplus* (Saito et al. 2005), R. *sanguineus* (Oliveira et al. 2005; Sanches et al. 2012a), A. *triste* (Oliveira et al. 2006), A. *brasiliense* (Sanches et al. 2010), A. *rotundatum* (Sanches et al. 2012b), A. *varium* (Sanches et al. 2014)d *annulatus* (Kanapadinchareveetil et al. 2015, 2019).

In the present study, scanning electron microscopical examination of the ovary of fed *H. dromedarii* immersed in ethanolic extract of *C. colocynthis* (100 mg/mL) showed stretched ovary with deformed oocytes having indistinct boundaries, and wrinkled, eroded surfaces.

Microscopical examinations of the tick ovary revealed that the oocytes of the treated females exhibited changes that affected their growth and development when the plant extract was applied after engorgement. The overall damage observed included great deformity in the ovarian wall and a decrease in the number of oocytes. According to Oliveira et al. (2005), the ovarian epithelium is involved in the oocyte’s attachment and transportation of the yolk elements from the hemolymph to the oocytes. Thus, it was suggested that the damage to the ovarian wall affected the metabolism and development of oocytes.

There was lysis of cell organelles, especially mitochondria, and the appearance of large cytoplasmic vacuoles after treatment. It was suggested that these vacuoles are autophagic and are responsible for the degradation of organelles that have been damaged by the action of plant extract. Damaged mitochondria suggested a decrease in ATP production, causing an effect on respiratory metabolism (Iturbe-Requena et al. 2020) and all cell activities (Alberts et al. 2010) interfering in the development of these cells and even preventing the continuation of vitellogenesis (Oliveira et al. 2017). The energy loss was also reported by Denardi et al. (2012) and Remedio et al. (2015) in *R. sanguineus* treated with neem extract and neem oil, respectively. Carvalho and Recco-Pimentel (2007) explained the presence of vacuoles as structures found mainly in cells where degradation and recycling of damaged portions and cytoplasmic organelles take place. They may be a result of a detoxification mechanism to isolate interfering substances or organelles without functions (Arnosti et al. 2011a, b) or caused by increased permeability of the oocyte membrane (Vendramini et al. 2012; Konig et al. 2019).

In the present study, the nucleus suffered from an abnormal appearance with a vacuolated nucleolus reflecting the ability of the plant extract to reach it. The nucleolus plays a pivotal role in the synthesis of proteins (Scheer and Hock 1999) that are important for oocyte development (Konig et al. 2020). The presence of a vacuolated nucleolus led to degeneration of genetic material (Remedio et al. 2015), and thus cell death (Denardi et al. 2011; Vendramini et al. 2012; Remedio et al. 2014), preventing the completion of vitellogenesis (Oliveira et al. 2019).

Fragmentation and decrease in the number of yolk granules were observed after treatment. As the vacuolated areas increase, the areas occupied by the yolk granules decrease, which affect the vitellogenesis, the formation of embryos and also impair the offspring’s survival. Iturbe-Requena et al. (2020) reported that the yolk granules store the nutrients of embryos, their damage would affect the survival of the progeny.

Damaged microvilli were clearly demonstrated in the present study between cell membrane and tunica propria. This may be due to the result of extract penetration through the egg that ruptures cell membrane and damages the microvilli. This considered a defense mechanism resulted in decrease in the surface area adhering to the haemolymph and/or decrease in the exogenous uptake of yolk precursors explaining the delay or inhibition of vitellogenesis (Marzouk et al. 2020).

A separation between tunica propria and the cell membrane was observed that led to disruption in the formation of chorion and demonstrated the capability of the plant extract to damage the first protective cell barrier. Vitellogenesis was inhibited or delayed where stage V oocytes were not formed during the examined period. Vitellogenin is a protein whose concentration increases with female engorgement, and regulation occurs through its uptake by oocytes (Seixas et al. 2010). The *C. colocynthis* extract interfered with hormonal regulation, leading to a decrease in the production of vitellogenic elements and reducing the release of vitellogenin through the inhibition of ecdysteroid hormones.

The chorion allows the oxygenation of the embryo, protects oocytes, and regulates the entry and exit of elements (Oliveira et al. 2016). The disruption of this membrane would expose oocytes to the hemolymph and allow toxic compounds to penetrate the oocyte (Arnosti et al. 2011b). This caused an increase in vacuoles and yolk granules lost their round shape, became irregular and began to lyse which led to major changes in the cytoplasm of oocytes (Arnosti et al. 2011b). As the chorion deposition would start in oocyte stage III and be completed in stage V, oocytes I and II are more susceptible to the intake of toxic compounds (Oliveira et al. 2019; Souza et al. 2019), which explain the damage to oocytes observed in our results. In some cases, the chorion is not able to prevent the total absorption of the product due to the damage caused to its structure, which can be confirmed by the presence of folds and rupture in the membranes of these cells. Thus, the chorion loses its original protective function, allowing the endocytosis of the extract by the oocytes and consequent harmful action in the cells (Vendramini et al. 2012).

In studies evaluating the effect of botanical acaricides on ticks, morphophysiological alterations of the oocytes during their development were observed (Nwanade et al. 2020). The fully engorged tick females reached their critical weight and then detached from the host (Kanapadinchareveetil et al. 2019). After detachment, the nervous system of the tick had no influence on the reproductive system (Weiss and Kaufman 2001). Botanical acaricides can affect the morphophysiology of reproductive organs (Konig et al. 2020). In blood-feeding arthropods, blood provides a rich source of proteins for vitellogenesis and egg production (Horn et al. 2009). During digestion of the blood, a large amount of lipids is secreted into the hemolymph and will be taken up by the growing oocytes to produce vitellogenin (Atella et al. 2005). According to Balashov (1983) and Xavier et al. (2018), vitellogenin is also synthesized in the tick gut for yolk production.

Despite the presence of a defense mechanism exhibited by oocytes to overcome the effect of extract, these cells were not able to recover this damage leading to cell death and vitellogenesis stopped. It is suggested that plant extract could affect different stages of oocytes in the tick ovary in two ways, directly by absorption through the tick integument and transport through the hemolymph until reaching the oocytes, causing their damage, or indirectly entering the gut through mouthparts, then affecting either gut cells or the process of blood digestion, leading to disturbances in vitellogenin production which prevent vitellogenesis.

## Conclusion

Histological observations of fed female *Hyalomma dromedarii* ovaries, after the treatment with ethanolic extract of *Citrullus colocynthis*, provided evidence that it suffered from extensive damage of different types of oocytes. Destruction of the internal organelles of oocytes and/or inhibition of vitellogenesis were demonstrated. This is the first histological study that points to the damage in *H. dromedarii* ovaries following treatment with ethanolic extract of *C. colocynthis*. The data presented suggest that this plant extract can be used as a promising agent for tick control generally including *H. dromedarii*.

## Data Availability

None.
